# Lights out: PP2C phosphatases reset phototropin signaling in darkness

**DOI:** 10.1093/plcell/koag049

**Published:** 2026-02-26

**Authors:** Barbara Bourgade

**Affiliations:** Assistant Features Editor, The Plant Cell, American Society of Plant Biologists, USA; Department of Organismal Biology, Uppsala University, 752 36 Uppsala, Sweden

Light is a highly dynamic environmental cue, and plants have evolved finely tuned signaling systems to translate changes in light conditions into appropriate physiological and developmental responses. The blue light–responsive kinases phototropin 1 (phot1) and phototropin 2 (phot2) regulate multiple adaptive responses, including chloroplast movement, stomatal opening and hypocotyl phototropism, which together optimize photosynthetic efficiency (Okajima 2016). Hypocotyl phototropism requires a lateral auxin gradient that drives asymmetric growth and curvature of the hypocotyl. This process involves the signaling component NON-PHOTOTROPIC HYPOCOTYL 3 (NPH3), which interacts with phot1 at the plasma membrane. NPH3 belongs to the NPH3/RPT2-like (NRL) protein family and is rapidly and reversibly phosphorylated by phot1 upon blue light activation (Reuter et al. 2021; Sullivan et al. 2021). This triggers the relocalization of NPH3 from the plasma membrane into cytosolic condensates, a process important for hypocotyl phototropism. At the molecular level, phot1 phosphorylates NPH3 at serine 744 (S744), promoting 14-3-3 protein binding and driving NPH3 dissociation from the plasma membrane, thereby weakening its interaction with phot1. Dephosphorylation of S744 is necessary to restore NPH3 plasma membrane localization (Reuter et al. 2021; Sullivan et al. 2021) and re-establish the phot1-NPH3 complex. However, the molecular mechanisms and regulatory components responsible for this recycling step remain poorly understood.

In recent work, Stuart Sullivan and colleagues (Sullivan et al. 2026) identify 2 plasma membrane-associated clade L TYPE 2C PROTEIN PHOSPHATASES (PP2Cs), PP2C19 and PP2C35, as the enzymes jointly responsible for dephosphorylating NPH3 at serine 744 in *Arabidopsis thaliana*. Mutants lacking PP2Cs from clades E and D exhibited dephosphorylation kinetics at S744 comparable to the wild-type following dark exposure, whereas a *pp2c19-1* mutant showed delayed dephosphorylation, a defect that was further exacerbated in the double mutant *pp2c19-1/35-1*. The authors next demonstrated that PP2C19/35-mediated dephosphorylation promoted the relocalization of NPH3, which suggests a functional role for these 2 phosphatases in recycling NPH3 back to the plasma membrane. Furthermore, both *pp2c19-1* and *pp2c19-1/35-1* mutants displayed defects in hypocotyl phototropism under blue light, while transgenic expression of *PP2C19* and *PP2C35* in these 2 mutants restored phototropic curvature. Although hypocotyl length was unaffected in the *pp2c19-1/35-1* double mutant, defective gravitropism was observed specifically in the hypocotyl. This phenotype was accompanied by reduced seedling emergence when seeds were buried in soil (see Fig. 1A and B), reflecting compromised directional growth during early seedling establishment.

**Figure 1:**
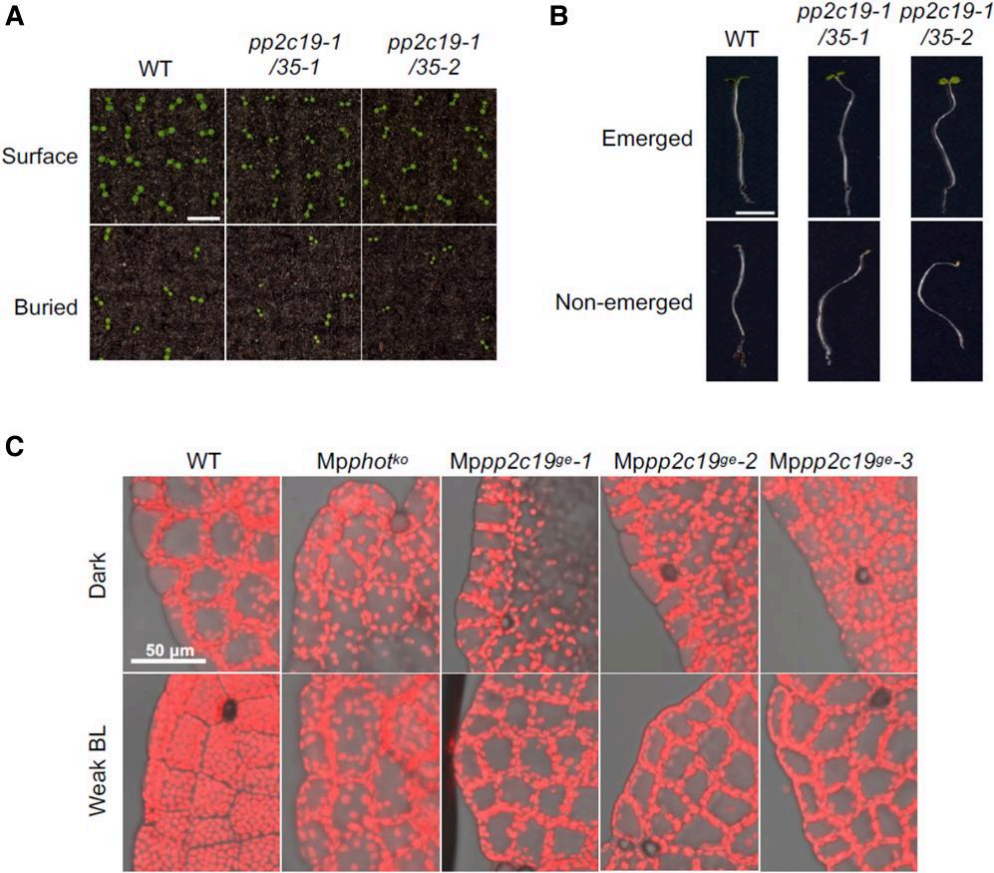
*Loss of PP2C19 and PP2C35 phosphatases causes developmental defects.* (A) Impaired seedling emergence in an *A*. *thaliana pp2c19-1/35-1* double mutant. The proportion of emerged seedlings was significantly reduced in the mutant strain when seeds were buried under the soil surface. *Surface*: seeds sown on the soil surface; *Buried*: seeds buried 1 cm into the soil; (B) In addition to reduced emergence, the *pp2c19-1/35-1* seedlings exhibited a curved phenotype. (C) Reduced chloroplast (red) accumulation in *M. polymorpha* mutants lacking *PP2C19* (Mp*pp2c19ge*) in low blue light (BL). Adapted from Sullivan et al. 2026, Figure 6A; 6C and Figure 11C.

Interestingly, targets responsive to PP2C19 and PP2C35 extended to ROOT PHOTOTROPISM 2 (RPT2), an NRL protein that interacts with NPH3 in phototropic signaling. Serine 591 of RPT2, which is phosphorylated by phot1 in response to blue light, was rapidly dephosphorylated in darkness by PP2C19/35—a response that was abolished in the *pp2c19-1/35-1* double mutant. Moreover, RPT2 protein abundance was increased in the *pp2c19-1/35-1* mutant without a corresponding rise in transcript levels, indicating that PP2C19 and PP2C35 promote proteasome-dependent degradation of RPT2 in the absence of blue light. This study further identified a phot1-dependent phosphorylation site at serine 602 (S602) in NRL PROTEIN FOR CHLOROPLAST MOVEMENT 1 (NCH1), another member of the NRL family that coordinates chloroplast movement alongside RPT2 under low light. Notably, S602 was also dephosphorylated by PP2C19 and PP2C35, suggesting that these phosphatases act on a broader set of phot1 targets. Loss of *PP2C19* and *PP2C35* resulted in impaired chloroplast accumulation under low blue light, implicating PP2C19/35-mediated dephosphorylation of RPT2 and NCH1 in this response. Importantly, a similar defect was observed in *Marchantia polymorpha pp2c19* mutants (see Fig. 1C), pointing toward a conserved function of these clade L PP2Cs across land plants.

In conclusion, 2 clade L PP2Cs act as central regulators of the dynamic dephosphorylation of phot1-mediated phosphorylated sites across multiple signaling components. By coordinating the regulation of 3 members of the NRL protein family—NPH3, RPT2, and NCH1—PP2C19 and PP2C35 are essential for hypocotyl phototropism and gravitropism as well as chloroplast accumulation in response to blue light. Uncovering additional PP2C19/35 targets and elucidating how these phosphatases are regulated will be crucial to further unravel the complexity of light-responsive signaling networks controlling plant development.

## Recent related articles in *The Plant Cell:*

- Hao et al. (2023) demonstrated that pure green light stimulates hypocotyl elongation in *Arabidopsis thaliana* by activating the brassinosteroid signaling pathway through enhancing the DNA-binding activity of the master transcription factor BRI1-EMS-SUPPRESSOR 1.
- Kong et al. (2024) identified CHLOROPLAST UNUSUAL POSITIONING 1 as a key actin polymerization factor involved in the assembly of chloroplast-associated actin filaments in a blue light- and phototropin-dependent manner, thereby enabling light-regulated chloroplast movement in *Arabidopsis thaliana*.
- Lopez Vasquez et al. (2023) showed that S-acylation of conserved cysteine residues within motif C of PHYTOCHROME KINASE SUBSTRATE 4 is essential for its localization at the plasma membrane, where it regulates phototropism and hypocotyl gravitropism.
- Xiao et al. (2025) identified KINESIN-LIKE CALMODULIN-BINDING PROTEIN-INTERACTING PROTEIN KINASE and its paralogue KIPKL1 as plasma membrane-associated regulators of negative hypocotyl gravitropism and obstacle avoidance that activate PIN-mediated auxin transport to prevent excessive hypocotyl bending.

## Data Availability

No new data was generated.
